# Correction: Davies et al. Impact of Refinements to Handling and Restraint Methods in Mice. *Animals* 2022, *12*, 2173

**DOI:** 10.3390/ani13142275

**Published:** 2023-07-12

**Authors:** Jennifer R. Davies, Dandri A. Purawijaya, Julia M. Bartlett, Emma S. J. Robinson

**Affiliations:** School of Physiology, Pharmacology & Neuroscience, Biomedical Sciences Building, University of Bristol, University Walk, Bristol BS8 1TD, UK

## Error in Figure/Table

In the original publication [1], there was a mistake in Table 3 as published. The table incorrectly described the methods for the quantification of struggling behaviour. The corrected Table 3 appears below.

In the original publication [1], there was a mistake in Figure 4 as published. The colour of the bars for panel F were the wrong way around, and there was an error in the description of the groups in the legend. The corrected Figure 4 and legend appears below. 

The authors would like to apologize for any inconvenience to the readers caused by these errors. The authors state that the scientific conclusions are unaffected. This correction was approved by the Academic Editor. The original publication has also been updated.

## Figures and Tables

**Figure 4:**
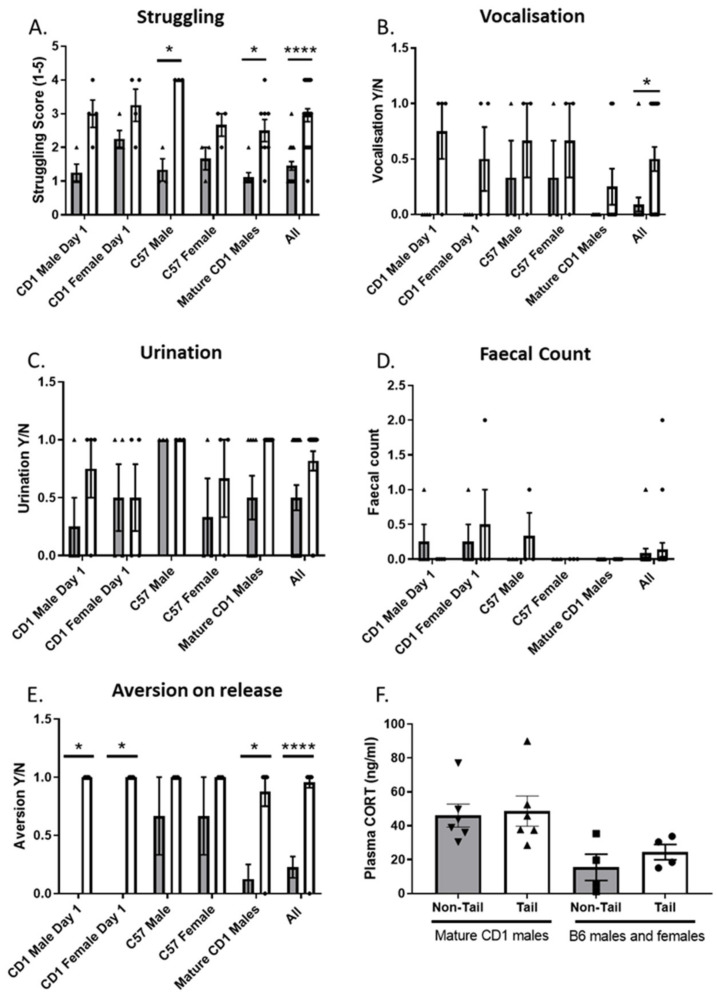
Effect of handling method on overt behaviours following different restraint methods. (**A**) Mice handled by non-tail restraint showed lower scores for struggling (Kruskal–Wallis ANOVA *p* < 0.0001), (**B**) Mice handled by non-tail restraint showed lower occurrence of vocalization (Kruskal–Wallis ANOVA *p* < 0.0121), (**C**) lower occurrence of urination, (**D**) lower average faecal count, (**E**) lower scores for aversion to release (Kruskal–Wallis ANOVA *p* < 0.0001). For groups CD1 males *n* = 16 (tail = 8, arm = 8), CD1 females *n* = 16 (tail = 8, arm = 8), B6 males *n* = 6 (tail n = 2, arm *n* = 4), B6 females *n* = 6 (tail = 4, arm *n* = 2), CD1 mature males *n* = 8 (tail = 4, arm = 4). (**F**) No effects on CORT were observed CD1 males (tail = 4, arm = 4) and B6 male and female (tail *n* = 6, arm *n* = 6). Data shown as mean ± S.E.M. (* *p* < 0.05, **** *p* < 0.0001). Grey bar = non-tail restraint, White bar = tail restraint.

**Table 3:** Visual overt behavioural observations during non-tail and the conventional tail restraint methods.

Visual Observations
Struggling effort to be released from grip (1) No struggling once restrained (2) Slight struggling for a short period of time (3) Slight struggling throughout/moderate struggling for a short period (4) Moderate struggling throughout/severe struggling for a short period (5) Severe struggling throughout Vocalisations made during grip Yes = 1, No = 0 Urination during or after grip Yes = 1, No = 0 Escape behaviour (indicated by running or avoiding hands) Yes = 1, No = 0
